# Editorial: New insights on B cell development in poultry

**DOI:** 10.3389/fimmu.2026.1899604

**Published:** 2026-06-15

**Authors:** Gregory Todd Pharr, Sonja Härtle, Nandor Nagy

**Affiliations:** 1 College of Veterinary Medicine, Mississippi State University, Mississippi, MS, United States; 2 Department of Veterinary Sciences, AG Immunology, Ludwig-Maximilians-Universität München, Planegg, Germany; 3 Department of Anatomy, Histology and Embryology, Faculty of Medicine, Semmelweis University, Budapest, Hungary

**Keywords:** B cell development, bursa of Fabricius, bursal microenvironment, humoral immunity, Ig-gene conversion, lymphoid organogenesis

The bursa of Fabricius (BF) is a gut-associated primary lymphoid tissue guiding B cell development in chickens. In embryogenesis, a single wave of prebursal stem cells colonize the nascent follicles of the BF. Within the follicles the cells proliferate and then initiate repertoire development by immunoglobulin gene conversion (GC). At hatching, bursal B cells migrate across the membrane of the follicular medulla into the outer cortex of the follicle. In the cortex the B cells continue proliferation and repertoire development. B cells are exported from the cortex to the periphery upon completion of the developmental program(s).

This Research Topic includes four original research articles and one review to highlight recent advances in chicken B cell development and peripheral B cell biology. Two articles will present novel mechanisms of BF lymphoid follicle development, as well as the mechanisms behind B cell migration within the follicles and for export to the periphery. As B cell development involves multiple levels of signaling, considerable progress is reported for B cell surface proteins involved in cell communication within the BF follicles. The genetic mechanisms governing GC in B cell receptor (BCR) repertoire development will be reviewed including the use of these genetic processes in intriguing new technologies. This Topic also includes new research evaluating changes in the peripheral BCR repertoire in response to infection or vaccination. New findings show that the chicken BCR repertoire, as in mammals, consists of both public and private specificities, which can be observed with antigen challenges.

The embryonic BF is colonized by granulocyte precursors, macrophages, and bursal secretory dendritic cells (BSDC) prior to prebursal stem cells. The presence of the BSDC in the nascent follicles is critical for recruitment of prebursal stem cells and has been considered to induce follicle development as well. However, previous studies have revealed the development of a few follicles after testosterone mediated inhibition of the BSDC, leading Szőcs et al. to propose that a different cell type may initiate folliculogenesis. Comprehensive phenotype analysis revealed a cell population with monoclonal antibody EIV-E12 which lacked myeloid, lymphoid or BSDC markers. Elegant studies established that the EIV-E12+ cells preceded the BSDC into the BF epithelium, and duck-chick chimera techniques showed that follicle development did not occur in the absence of EIV-E12+ cells, thus establishing the EIV-E12+ cells as a novel lymphoid tissue inducer cell for bursal folliculogenesis.

A CXCR4/CXCL12 axis controls the entry of CXCR4^hi^ prebursal stem cells into the bursal mesenchyme, followed by recruitment into the nascent follicles, where BSDC are the source of CXCL12. However, at hatching, B cells start migrating from the medulla to the cortex, and then to the periphery by ill-defined mechanisms. Soós et al. present a new model for function of the cortex. The novel CXCL12-abundant reticular cells identified in the outer region of the cortex recruit CXCR4^hi^ medullary B cells across the cortico-medullary border at tenascin-C free areas between the capillaries. At the outer region of the cortex B cells proliferate and undergo additional rounds of GC. Surrounding the capillaries, the tenascin-C network contains CXCL12 and displays an inhibitory effect on the migration of CXCR4^hi^ B cells. However, once cortical B cells complete their developmental program, expression of CXCR4 is reduced, facilitating B cell export, a developmental mechanism previously observed in mammalian bone marrow.

Many of the cell surface proteins involved in cellular communication during bursal B-cell development remain unknown. However, one strong candidate for cellular communication is detailed in the original article by Funk, whose laboratory has conducted an extensive characterization of the chB6 alloantigen, a pan – B cell marker. The chB6 protein contains two Ig-like extracellular domains like the mammalian CD2/SLAM family including the SH3 binding consensus site in the cytoplasmic domain. The signals through the mammalian CD2/SLAM family are important for modulating lymphocyte cell fate decisions. Indeed, the cytoplasmic domain of chB6 recruits an initiator caspase in chicken B cells, providing a mechanism for the long-standing observation that cross-linking chB6 on bursal B cells induces apoptosis *in vitro* and emphasizes a substantial role for chB6 in delivering fate determining signals during bursal B cell development.

Seo et al. give a thorough description of the genetic mechanisms which can result in either GC or somatic hypermutation (SHM). Utilizing these mechanisms, Seo’s group developed the novel autonomously diversifying library (ADLib) system using the chicken bursal B cell line DT40 to generate a substantial library of antibody specificities. Importantly, the ADLib system has also been adapted to generate humanized antibodies. In DT40, SHM is enhanced when a gene critical for GC is deleted. Using this concept, Seo and coworkers inserted the biological marker green fluorescent gene into the light chain locus. Several rounds of SHM resulted in DT40 cells exhibiting an increased fluorescence, indicating protein optimization. Significantly, this system has also been used to increase the affinity of single-chain variable fragments for use in diagnostics or as therapeutic antibody-drug conjugates.

Outside of standard serology, little is known about the changes in the avian BCR repertoire in response to antigens. Dascalu et al. report a thorough evaluation of the IgM and IgY clones in chickens vaccinated or infected with avian influenza virus. The authors provide novel evidence for convergent humoral immune responses in poultry, with many clones being shared among different individuals. The authors suggest that the identification of public BCRs could reflect biases during the development of the pre-immune repertoire by GC or for certain CDR3 sequences. The convergent responses were found to be influenced by route of exposure (vaccination or infection), as well as the number of exposures. Fundamentally, using the approach detailed by Dascalu et al. to understand how a population of poultry (e.g., broiler line) responds to a vaccine candidate could help guide vaccine optimization during vaccine development.

In conclusion, our Research Topic highlights the development of the BF, bursal B cell development, and advances our understanding of peripheral B cell responses to infection or vaccination. We hope this Topic stimulates further research in avian immunology, utilizing the phenomenal technology currently available to the biological sciences.

